# Prediction of healthcare utilization following an episode of physical therapy for musculoskeletal pain

**DOI:** 10.1186/s12913-018-3470-6

**Published:** 2018-08-20

**Authors:** Trevor A. Lentz, Jason M. Beneciuk, Steven Z. George

**Affiliations:** 1 Duke Clinical Research Institute, Duke University, 2400 Pratt Street, Durham, NC 27705 USA; 2 Department of Physical Therapy, College of Public Health & Health Professions, University of Florida, Box 100154, UFHSC, Gainesville, FL 32610-0154 USA; 3 Brooks Rehabilitation Clinical Research Center, 3901 University Blvd. South, Suite 103, Jacksonville, FL 32216 USA; 4 Duke Clinical Research Institute, Department of Orthopaedic Surgery, Duke University, 2400 Pratt Street, Durham, NC 27705 USA

**Keywords:** Screening, Psychological distress, Multimorbidity, Value, Treatment monitoring

## Abstract

**Background:**

In the United States, value-based purchasing has created the need for healthcare systems to prospectively identify patients at risk for high healthcare utilization beyond a physical therapy episode for musculoskeletal pain. The purpose of this study was to determine predictors of pain-related healthcare utilization subsequent to an index episode of physical therapy for musculoskeletal pain.

**Methods:**

This study assessed data from the Optimal Screening for Prediction of Referral and Outcome (OSPRO) longitudinal cohort study that recruited individuals with a primary complaint of neck, low back, knee or shoulder pain in physical therapy (*n* = 440). Demographics, health-related information, review of systems, comorbidity and pain-related psychological distress measures were collected at baseline evaluation. Baseline to 4-week changes in pain intensity, disability, and pain-related psychological distress were measured as treatment response variables. At 6-months and 1-year after baseline evaluation, individuals reported use of opioids, injection, surgery, diagnostic tests or imaging, and emergency room visits for their pain condition over the follow-up period. Separate prediction models were developed for any subsequent care and service-specific utilization.

**Results:**

Subsequent pain-related healthcare utilization was reported by 43% (*n* = 106) of the study sample that completed the 12-month follow-up (*n* = 246). Baseline disability and 4-week change in pain intensity were important global predictors of subsequent healthcare utilization. Age, insurance status, comorbidity burden, baseline pain, and 4-week changes in pain intensity, disability and pain-related psychological distress predicted specific service utilization.

**Conclusion:**

In those completing follow up measures, risk of additional pain-related healthcare utilization after physical therapy was best predicted by baseline characteristics and 4-week treatment response variables for pain intensity, disability and pain-related psychological distress. These findings suggest treatment monitoring of specific response variables could enhance identification of those at risk for future healthcare utilization in addition to baseline assessment. Further study is required to determine how specific characteristics of the clinical encounter influence future utilization.

## Background

Musculoskeletal pain is a prevalent and costly health condition with far-reaching public health consequences including chronic pain, disability and opioid-related addiction [1]. Clinical practice guidelines now recommend non-pharmacological treatment as frontline management for musculoskeletal pain, which will lead to increased utilization of services such as physical therapy [1–3]. Physical therapy is effective for improving disability and reducing costs associated with many musculoskeletal pain conditions [4–9]. However, pain-related healthcare utilization beyond the physical therapy episode (e.g. subsequent use of surgery, injection, opioids, etc.) may indicate suboptimal treatment response, the presence of more complex needs, or unwarranted escalation of care. Downstream healthcare utilization is not often considered as an outcome of care or indication of treatment effectiveness for musculoskeletal pain. But the importance of identifying risk for additional utilization has emerged due to the growth of cost-sharing and capitated payment models, particularly in the United States (US). As a result, many US health care services organizations have begun to prioritize early identification of individuals at risk for downstream healthcare use at the onset of treatment [10, 11]. Early risk assessment allows systems to deliver greater value by 1) focusing limited health care resources towards patients who are most in need, and 2) identifying those who may require coordination of multiple providers and services to optimize outcomes.

Prospective identification of risk for high subsequent healthcare utilization is a different approach to outcomes prediction for musculoskeletal pain [12, 13] and one that has not been evaluated in physical therapy settings in the US. Most existing outcomes prediction models focus on pain and disability endpoints [12–14]. They also concentrate on condition-specific and psychological predictors, with less attention to factors that could influence healthcare utilization more directly [15–17]. These factors include insurance, comorbidities, symptoms unrelated to the pain condition, and treatment response. As a result, predictors of pain-related healthcare utilization beyond physical therapy are unknown. A better understanding of these predictors will have significant implications for future healthcare pathway development. For instance, an influence of modifiable factors like pain-related psychological distress might imply the need to build clinical pathways that address those factors directly through physical therapist provided intervention. Additionally, understanding the relative predictive capabilities of baseline versus change estimates for modifiable factors would clarify whether prediction is improved by routinely assessing outcomes during the course of treatment (i.e. treatment monitoring) [18].

This study was undertaken in a nationwide, US cohort of patients receiving outpatient physical therapy for a primary complaint of knee, shoulder, back or neck pain. The primary aim of the analysis was to predict incidence of additional pain-related healthcare utilization in the year following the episode of physical therapy for musculoskeletal pain. We considered factors not commonly assessed in outcomes prediction for musculoskeletal pain, like insurance, comorbidities, and treatment response, as well as those more often associated with pain-related outcomes (e.g. psychological distress). This project will lead to the development of potentially novel outcome prediction models for this population in a common, non-pharmacological US healthcare setting. The results of this study will be particularly important in value-based payment settings where enhanced clinical decision-making drives treatment effectiveness and system efficiency.

## Methods

### Dataset and patient population

This study used data from the Orthopedic Physical Therapy – Investigative Network’s (OPT-IN) Optimal Screening for Prediction of Referral and Outcome (OSPRO) validation cohort study, a longitudinal prospective study of individuals with knee, shoulder, back or neck pain seeking Physical Therapy in the US. A convenience sample was recruited from December 2014 and December 2015 by participating OPT-IN clinics. The OPT-IN clinics that participated in data collection represented multiple geographic regions in the US including the Mideast, Southeast, Great Lakes, Rocky Mountain States and Far West, with an attempt to balance recruitment between urban and rural settings over the entire OPT-IN network. Physical therapists practicing in these clinics identified eligible participants at initial evaluation and directed them to a secure study website for the informed consent process and baseline self-report assessment. Eligibility criteria have been thoroughly reported elsewhere [19] and were intentionally broad to develop a cohort that was generalizable to those seeking physical therapy for common musculoskeletal conditions in the US. Participants completed follow-up self-reported assessments on the study website at 4 weeks, 6 months and 12 months. Participants were notified of a pending assessment by an email that directed them back to the study website to complete their follow-up assessment. For additional details of the dataset and cohort, readers are directed to the published cohort profile [19].

The primary aim of the OSPRO cohort study was to develop and validate review of systems (i.e. evidence of systemic involvement) and yellow flag (i.e. pain-related psychological distress) screening tools for use in outpatient orthopedic physical therapy settings. These screening tools, once validated and refined for clinical decision making, may improve the value of care delivery by accurately identifying individuals who 1) are appropriate for referral to other providers for management of non-musculoskeletal symptoms, and/or 2) would benefit from enhanced, psychologically-informed physical therapy. Early identification of individuals most appropriate for these modified pathways of care has the potential to reduce wasteful downstream health care utilization, limit the risk of unwarranted and costly care escalation, and improve clinical outcomes. Results of the primary analyses examining the predictive ability of the OSPRO tools for pain, disability, health status, and comorbidity outcomes have been previously published [20]. Pre-planned secondary analyses included prediction of persistent pain state [21] and this current analysis predicting future healthcare utilization. All subjects consented to participation in the study and ethics approval was granted by the University of Florida Institutional Review Board.

### Healthcare utilization predictors

We collected potential predictors by self-reported questionnaires at initial evaluation using an online study website. Participants were directed back to the study website 4 weeks following initial evaluation to again complete questions on pain intensity, disability, and pain-related psychological distress. Change in pain intensity, disability, and pain-related psychological distress from baseline to 4 weeks were modeled as treatment response variables and included as potential predictors.

### Sociodemographic and health-related information

Participants completed a standard intake questionnaire form previously used in our clinical studies that assessed age, sex, race, and insurance provider type. This questionnaire also assessed health-related variables included anatomical region of primary pain complaint (low back, neck, shoulder, or knee) and whether the patient had undergone surgery for their primary pain complaints (yes or no). Due to small cell sizes for certain categories, race was dichotomized as white or non-white. For insurance type, participants were asked to choose one of the following options: private, public (Medicare and/or Medicaid), uninsured/self-pay, worker’s compensation, and other/commercial insurance. Among the study sample, we observed few with no insurance (*n* = 7) or worker’s compensation (*n* = 14). The study also included relatively few with ‘other/commercial insurance’ (*n* = 45). Within this group, informal assessment of these various plans suggested high heterogeneity of plan characteristics and coverage. Due to the small number of subjects in these individual insurance strata and to improve interpretability of results, we collapsed those reporting no insurance, worker’s compensation and other/commercial insurance into a single category (i.e., ‘Other’). Therefore, insurance type was categorized as private, public, or other (no insurance, worker’s compensation, or other/commercial insurance) for purposes of analysis.

### Pain-related clinical variables

Pain status was determined using established definitions that account for the duration of pain and activity limitations [22, 23] using the following two questions: 1) “How long have you been experiencing your current painful symptoms?” and 2) “Have you experienced ANY pain and activity limitations every day for the past 3 months?” Responses to question 1 of “greater than 90 days” or responses to question 2 of “Yes” were used to classify patients as having persistent pain at initial evaluation.

### Pain intensity

Pain intensity was assessed by the numerical pain rating scale (NPRS) ranging from “0” (no pain) to “10” (worst pain imaginable) [24–26]. Participants rated their current pain intensity, as well as their best (lowest) and worst (highest) pain intensity over the past 24 h. Current, best and worst pain ratings were averaged for purposes of analysis.

### Region-specific disability

Self-reported region-specific disability was assessed with the Neck Disability Index [27, 28], Oswestry Disability Questionnaire [29, 30], Quick Disability of Arm Shoulder and Hand [31] or International Knee Documentation Committee Subjective Knee Form [32] for cervical, low back, shoulder and knee pain, respectively. Region-specific disability measures were z-transformed for purposes of analysis, consistent with our prior work involving multiple anatomical regions [33].

### Comorbidities

#### Charlson comorbidity index (CCI)

The Charlson Comorbidity Index was used to measure the presence of chronic comorbid medical conditions [34]. It lists 19 medical conditions that participants are asked to indicate whether they “have ever been diagnosed with by a physician”. Conditions are weighted and added for an overall measure of comorbidity burden. The CCI has demonstrated good test-retest reliability (0.91) and positive but weak to modest correlations with medication use, hospitalizations, length of stay, total charges, and pharmacy and laboratory charges for older adults in general medical care and surgical care settings [35].

### Assessment tools

#### OSPRO Review of Systems tool (OSPRO-ROS)

The OSPRO-ROS is a review-of-systems screening tool for use in outpatient orthopedic physical therapy settings [36]. The OSPRO-ROS has demonstrated good concurrent validity with depression and a comprehensive 97-item battery of non-musculoskeletal symptoms (i.e., red flags). [36] Moderate to strong predictive capabilities of the OSPRO-ROS have been reported for persistence of pain, quality of life, and change in comorbidity 12 months following physical therapy in patients with musculoskeletal pain [20, 21]. The OSPRO-ROS includes standard symptom descriptors to aid with identification of systemic or non-musculoskeletal origins of musculoskeletal pain. It includes questions related to symptoms of the cardiovascular, gastrointestinal, endocrine, nervous, integumentary, pulmonary, and musculoskeletal systems. The full-length 23-item version of the OSPRO-ROS is capable of identifying 100% of positive red-flag responders (i.e. indicating “yes” to at least one systemic symptom on a questionnaire) in outpatient orthopedic physical therapy settings. [36] A shorter, 10-item version is also available that has been shown to identify approximately 95% of positive red-flag responders. For statistical analyses, the “yes” responses were added for each version and included in each model as a continuous independent variable.

#### OSPRO Yellow Flag tool (OSPRO-YF)

The OSPRO-YF is a yellow flag assessment tool that includes items from pain vulnerability domains (negative affect and fear-avoidance) and pain resilience domains (positive affect and self-efficacy) to aid with identification of pain-related psychological distress in outpatient orthopedic physical therapy settings [37]. The OSPRO-YF has good concurrent validity with pain intensity and region-specific disability [37] and is capable of predicting pain intensity, disability, quality of life and persistent pain 12 months following physical therapy in patients with musculoskeletal pain [20, 21]. The full-length OSPRO-YF has 17-items, however a shortened 10-item version is also available with an acceptable trade-off in accuracy. Like the OSPRO-ROS, the OSPRO-YF is designed for implementation into electronic medical record (EMR) systems to quickly and accurately identify risk for a variety of clinical outcomes [19]. For statistical analyses, a summary score was derived for each version by adding the item responses after reverse-scoring items 2, 13, 14, 15 and 17 so that higher scores indicate higher pain-related psychological distress. The summary score was then included in each model as a continuous independent variable.

### Intervention

All physical therapy treatment was provided at the discretion of the treating clinician. The duration of the episode, the number of physical therapy visits, and individual treatment parameters (type, intensity, duration, frequency) were not collected for pragmatic reasons. In particular, clinical and utilization data are not commonly collected in a standardized format and would need to be extracted from disparate medical record databases across different health care systems to assess treatment. This was not feasible given the scope and design of this multisite survey-based study. However, instead of coding treatment type we included baseline-to-4 week change in pain intensity, region-specific disability, and OSPRO-YF scores in each model as measures of treatment response. In that manner the individual effects of the treatment received were included in the predictive models, without directly accounting for the type of treatment.

### Healthcare utilization outcomes

Self-reported health care utilization was assessed at 6- and 12-months following initial evaluation by online assessment. Questions were derived from previous population-based studies involving musculoskeletal pain that have used survey methods for follow-up assessment [22, 23]. Study participants were asked whether they used any of the following healthcare services for their primary musculoskeletal pain complaint in the time following their physical therapy treatment:

1. Opioid painkillers (eg. Vicodin, Lortab, Hydrocodone, Fentanyl, Percocet, Oxycontin, Oxycodone, tramadol, Ultram, Diludid, etc)
2. Injections
3. Surgery
4. Diagnostic tests or Imaging (eg. xray, MRI, CT scan, nerve conduction test, etc.)
5. Emergency room visits

“Yes” responses were followed by questions regarding the quantity of services utilized (i.e. number of opioid painkillers, number of diagnostic tests or number of emergency room visits). All utilization questions were answered on a categorical scale (0, 1, 2–5, 5–10, or > 10) indicating the quantity of a particular service received during the applicable follow-up timeframe. At 6-month follow-up, study participants reported their use of services for the previous 2 months, allowing a timeframe of 4 months from initial evaluation for them to complete physical therapy. At 12-month follow-up, study participants reported their use of services over the previous 6 months since their last survey. This method provided an 8-month overall follow-up period after physical therapy and two follow-up points were included to minimize recall bias.

### Statistical analysis

All data analyses were preformed using SPSS Version 22.0 (IBM Corp., Armonk, NY). We developed models to separately predict over the course of the entire follow-up period: 1) the dichotomous outcome of no healthcare utilization versus any healthcare utilization and 2) the utilization of specific services. We decided to develop separate models since each outcome predicted by these models might have unique future policy implications. For instance, those who utilize no additional services might represent a “low risk” group for which physical therapy alone might be particularly appropriate. Predicting use of specific services would inform policy where reduction of specific services is a high priority, such as utilization of opioids or unnecessary use of emergency room services.

All prediction models used the following hierarchical design, which is similar to prior analyses in this cohort [20, 21]:

- Block 1: age, sex, race, anatomical region of pain, insurance, chronicity of pain, surgery for current condition (yes/no), Charlson comorbidity index, baseline disability, baseline pain intensity.
- Block 2: 10-item OSPRO-YF and 10-item OSPRO-ROS at baseline.
- Block 3: Remaining items from the OSPRO-YF (+ 7 items) and OSPRO-ROS (+ 13 items). These were included to determine whether full-length versions of the tools provided better prediction over shortened versions.
- Block 4: Baseline-to-4 week change in pain intensity, region-specific disability, and OSPRO-YF scores. Early changes in these variables may be associated with improved prediction of outcomes over baseline variables alone [38]. This approach modeled change in these variables as a measure of treatment response and allowed us to assess the relative value of treatment monitoring for the prediction of healthcare utilization outcomes.

For the first analysis, binary logistic regression was used to determine predictors of any healthcare utilization following physical therapy, with the dependent variable defined as reporting one or more utilization events for any of the potential healthcare services over the entire follow-up period. For analyses of specific services, utilization was dichotomized for each service. Specific service utilization over early (through 6 months) and late (6 months to 12 months) phases following physical therapy were collapsed to create a single dichotomous utilization indicator for each service over the entire study follow-up period. Any utilization of the service over that period was categorized as YES. Separate multivariate binary logistic regression models were then fitted for the dichotomous utilization indicator (i.e. YES or NO) of each healthcare service (e.g. opioid use, injection, imaging, surgery, and emergency room visits).

For all analyses, full hierarchical multivariate models were first fit to assess the unique contributions of each block. This approach allowed us to determine the relative contributions of baseline demographic and health-related variables, the newly developed OSPRO-ROS and OSPRO-YF tools, and response to treatment via time varying variables (e.g., pain intensity and region specific function). However, since our primary aim was to develop concise and accurate utilization prediction models for efficient assessment of risk, we then separately developed stepwise models using backward selection for each dependent variable to derive parsimonious prediction item sets. Parsimonious models were chosen as a more conservative approach to identifying individual predictors given the potential for overfitting full multivariate models because of high subject attrition. For stepwise models, the *p*-value threshold was 0.05 for entry and 0.10 for removal. Overall fit for each model was examined with Hosmer & Lemeshow test, chi-square and pseudo-R^2^ values (e.g. Nagelkerke) when appropriate. Comparison of adjusted odds ratios (OR) and 95% confidence interval (CI) were used to determine the relative strength of each predictor in parsimonious models. Multicollinearity was assessed using variance inflation factor (VIF) and tolerance, where VIFs < 10 and tolerances > 0.1 suggested no significant collinearity among independent variables [39].

### Planned sensitivity analyses for missing data

The electronic OPT-IN data collection forms required complete data from respondents before they were allowed to proceed to subsequent survey pages. Therefore, the occurrence of missing data for independent predictor variables was minimal (< 1% of sample). However, for subjects who were lost to follow-up, we planned two approaches to assess the potential influence of missing data on study outcomes. First, demographic and baseline health variables would be compared between those with complete follow-up at 1 year and those without follow-up at 1 year to identify any potential group differences related to completion of follow-up. Second, sensitivity analyses would be conducted by repeating each analysis using inverse probability of attrition weighting (IPAW). This propensity scoring approach accounts for attrition-related selection bias in longitudinal studies by more heavily weighting observations associated with a lower probability of study completion [40]. Thus, the resulting analysis is compensated for under-representation of subjects who are more likely to be lost to follow-up. IPAW produces smaller effect estimate biases than more conventional methods that adjust for baseline predictors of attrition [41]. Briefly, logistic regression will be performed to identify predictors of attrition using an opportunistic approach that optimizes model fit, with an area under the curve (AUC) target value of > 0.7. Demographic and baseline health variables that differ between follow-up status cohorts will be used as candidate variables for the regression model to derive weights. Then, inverse of predicted probabilities for remaining in the study will be used to weight observations, and all analyses will be repeated. Regression results using IPAW will be compared with those obtained from complete case only analyses to assess the potential influence of missing data on the findings and identify robust predictors. We will focus our interpretation on predictors that are consistent across complete case and IPAW models for each type of healthcare service as they are more robust and most likely to be reproduced in future studies.

### Power analysis

For logistic regression analyses, event-per-variable values of 10 or greater are suggested, since overfitting will weaken the probability that original findings will be reproduced in an independent sample [42, 43]. With 18 potential predictors, a sample of *n* = 180 reporting healthcare utilization at follow-up would be sufficient for the proposed analyses. However, this estimate is conservative. Other methods for determining sample size for prediction analyses suggest an overall sample size of *N* > 50 + 8*m (where *m* = number of independent variables) [44] or *N* > 104 + number of independent predictors [45, 46]. For these less conservative estimates, the projected study sample size is sufficient for the proposed analyses.

## Results

Four hundred and forty subjects were recruited at initial evaluation. Follow-up at 4 weeks was 75.0% (*n* = 330), at 6 months was 69.0% (*n* = 304) and at 12 months was 65.2% (*n* = 287). Baseline demographics and health-related characteristics for the full cohort, as well as those who did and did not complete all follow-up are presented in Tables 1, 2 and 3. Those who did not complete follow-up were younger, more likely to be non-white, had less than a college degree, were more likely to have had sudden symptom onset, had higher baseline pain intensity, and had higher baseline pain-related psychological distress measured by the OSPRO-YF. Only those with complete follow-up data at each time point were considered for prediction analyses (*n* = 246, 55.9%).

**Table 1:** Demographic information for the full cohort, and for those with complete and incomplete follow-up

Variable	Label	Full cohort at baseline (*n* = 440)	Completed follow-up (*n* = 246)	Did not complete follow-up (*n* = 194)	*p*-value ^a^
Demographic information					
Age	Mean ± SD	45.06 ± 15.82	46.59 ± 16.00	43.15 ± 15.43	0.02
	Median (min, max)	45 (18–75)	47 (18–75)	42 (18–74)	
Sex (1 missing)	Male	164 (37.3%)	85 (34.6%)	79 (40.7%)	0.20
	Female	275 (62.5%)	160 (65.0%)	115 (59.3%)	
Race (7 missing)	White	343 (78.0%)	200 (81.3%)	143 (73.7%)	0.05
	Non-white	90 (20.5%)	42 (17.1%)	48 (24.7%)	
Ethnicity (33 missing)	Hispanic or Latino	31 (7.0%)	20 (8.1%)	11 (5.7%)	0.36
	Not Hispanic or Latino	376 (85.5%)	211 (85.8%)	165 (85.1%)	
Education (6 missing)	Less than college graduate	161 (36.6%)	71 (28.9%)	90 (46.4%)	< 0.001
	College graduate or higher	273 (62.0%)	172 (69.9%)	101 (52.1%)	
Income (66 missing)	$35,000 or less	112 (25.5%)	62 (25.2%)	50 (25.8%)	0.30
	$35,000 to $70,000	106 (24.1%)	59 (24.0%)	47 (24.2%)	
	Greater than 70,000	156 (35.5%)	99 (40.2%)	57 (29.4%)	
Insurance (26 missing)	Private	273 (62.0%)	156 (63.4%)	117 (60.3%)	0.70
	Public	75 (17.0%)	46 (18.7%)	29 (14.9%)	
	Other	66 (15.0%)	36 (14.6%)	30 (15.5%)	
Geographic region	Southeast	275 (62.5%)	146 (59.3%)	129 (66.5%)	0.10
	Midwest	47 (10.7%)	23 (9.3%)	24 (12.4%)	
	West	98 (22.3%)	65 (26.4%)	33 (17.0%)	
	Northeast	20 (4.5%)	12 (4.9%)	8 (4.1%)	

^a^ Group comparisons with independent samples t-tests for continuous variables and chi-square tests for categorical variables

**Table 2:** Baseline health-related information for the full cohort, and for those with complete and incomplete follow-up

Variable	Label	Full cohort at baseline (*n* = 440)	Completed follow-up (*n* = 246)	Did not complete follow-up (*n* = 194)	*p*-value ^a^
Health-related information					
Anatomical region	Neck	98 (22.3%)	48 (19.5%)	50 (25.8%)	0.27
	Low Back	118 (26.8%)	66 (26.8%)	52 (26.8%)	
	Shoulder	107 (24.3%)	59 (24.0%)	48 (24.7%)	
	Knee	117 (26.6%)	73 (29.7%)	44 (22.7%)	
Symptom onset	Gradual	239 (54.3%)	146 (59.3%)	93 (47.9%)	0.03
	Sudden	138 (31.4%)	65 (26.4%)	73 (37.6%)	
	Traumatic	63 (14.3%)	35 (14.2%)	28 (14.4%)	
Duration of symptoms urgery for condition	Mean ± SD	398.58 ± 1715.80	379.79 ± 1999.77	423.01 ± 1259.33	0.80
	Median (min, max)	90 (0–29565)	90 (1–29565)	90 (0–10000)	
	Yes	83 (18.9%)	44 (17.9%)	39 (20.1%)	0.56
	No	357 (81.1%)	202 (82.1%)	155 (79.9%)	
Work-related injury (32 missing)	Yes	63 (14.3%)	30 (12.2%)	33 (17.0%)	0.36
	No	345 (78.4%)	198 (80.5%)	147 (75.8%)	
Chronicity	Acute	101 (23.0%)	65 (26.4%)	36 (18.6%)	0.05
	Chronic	324 (73.6%)	173 (70.3%)	151 (77.8%)	
Pain Intensity	Mean ± SD	4.22 ± 1.98	3.94 ± 1.72	4.58 ± 2.21	0.01
	Median (min, max)	4 (0–9.67)	4 (0–8)	4.5 (0–9.7)	
Disability	Mean ± SD	0 ± 1.0	−0.06 ± .97	0.08 ± 1.03	0.16
	Median (min, max)	−0.16 (−2.41–3.14)	−0.23 (−2.21–2.94)	−0.01 (−2.41–3.14)	
CCI	Mean ± SD	0.66 ± 1.47	0.68 ± 1.62	0.63 ± 1.25	0.76
	Median (min, max)	0 (0–13)	0 (0–13)	0 (0–8)	

*CCI* Charlson comorbidity index

^a^ Group comparisons with independent samples t-tests for continuous variables and chi-square tests for categorical variables

**Table 3:** Baseline OSPRO questionnaire scores for the full cohort and for those with complete and incomplete follow-up

Variable	Label	Full Cohort at baseline (*n* = 440)	Completed follow-up (*n* = 246)	Did not complete follow-up (*n* = 194)	*p*-value ^a^
OSPRO-ROS 10-item	Mean ± SD	2.68 ± 2.38	2.52 ± 2.24	2.89 ± 2.55	0.11
	Median (min, max)	2 (0–10)	2 (0–10)	2.5 (0–10)	
OSPRO-ROS + 13 items	Mean ± SD	1.25 ± 1.80	1.14 ± 1.52	1.38 ± 2.09	0.17
	Median (min, max)	1 (0–12)	1 (0–7)	1 (0–12)	
OSPRO-YF 10-item	Mean ± SD	17.43 ± 6.69	16.87 ± 6.46	18.15 ± 6.91	0.04
	Median (min, max)	17 (4–47)	16 (4–40)	17 (4–47)	
OSPRO-YF + 7 items	Mean ± SD	14.92 ± 5.51	14.41 ± 4.93	15.57 ± 6.12	0.03
	Median (min, max)	15 (3–34)	14 (3–28)	16 (3–34)	

*CCI* Charlson comorbidity index, *OSPRO-ROS* Review of systems screening tool, *OSPRO-YF* Pain-related psychological distress screening tool

^a^ Independent samples t-tests to assess group differences between those who did and did not complete follow-up

Overall, 43.1% (*n* = 106/246) of those with complete follow-up data utilized at least one healthcare service following the physical therapy episode. Distribution of utilization for specific services is provided in Table 4. For multivariate analyses, all VIFs were less than 10 and tolerance values greater than 0.1 suggesting no significant multicollinearity among independent variables.

**Table 4:** Frequency of healthcare utilization reported at 6-month and 12-month follow-up (*n* = 246)

Label	Utilization reported at 6-month follow-up					Utilization reported at 12 month follow-up					Dichotomous indicator for any healthcare utilization over entire follow-up	
Utilization volume	0	1	2–5	5–10	> 10	0	1	2–5	5–10	> 10	No	Yes
Opioids	209	18	16	1	2	204	19	16	7	0	201	45
Injection	212	17	14	2	1	217	17	12	0	0	206	40
Surgery	240	4	2	0	0	231	13	2	0	0	227	19
Diagnostic tests or imaging	183	40	22	1	0	188	26	28	4	0	172	74
Emergency room	237	7	2	0	0	232	11	2	1	0	228	18
Any care											140	106

### Full multivariate model performance

Overall performance for each full multivariate model is listed in Table 5. Block 1 (Demographic, clinical and comorbidity) consistently contributed to prediction of healthcare utilization and accounted for the greatest amount of variation in utilization outcome for all models. Block 4 (change scores for pain, disability, and OSPRO-YF) provided statistically significant contributions in all models except prediction of injection. Blocks including baseline OSPRO-YF and OSPRO-ROS, both short and long forms, did not predict utilization outcomes. Weighted models consistently outperformed their complete case analysis model counterparts with overall model pseudo-R^2^ values ranging from .337 (Any care) to .611 (Emergency room).

**Table 5:** Overall performance of full logistic multivariate regression models (*n* = 246)

Dependent variable	Any care	Opioids	Injection	Surgery	Diagnostic tests or imaging	Emergency room
*Complete Case*						
Block 1 Demographic, Clinical and Comorbidity	.258^**^	.274^**^	.292^**^	.226^*^	.250^*^	.400^**^
Block 2 OSPRO-YF (10 items) OSPRO-ROS (10 items)	.267	.294	.293	.234	.253	.404
Block 3 OSPRO-YF (+ 7 items) OSPRO-ROS (+ 13 items)	.275	.296	.315	.259	.271	.457
Block 4 4-week change (Pain, Disability, OSPRO-YF)	.337^*^	.424^**^	.353	.426^**^	.340^*^	.560^*^
*Inverse Probability of Attrition Weighted*						
Block 1 Demographic, Clinical and Comorbidity	.306^**^	.294^**^	.313^**^	.236	.304^**^	.430^**^
Block 2 OSPRO-YF (10 items) OSPRO-ROS (10 items)	.314	.317	.317	.250	.305	.435
Block 3 OSPRO-YF (+ 7 items) OSPRO-ROS (+ 13 items)	.321	.321	.334	.284	.321	.473
Block 4 4-week change (Pain, Disability, OSPRO-YF)	.382^*^	.448^**^	.373	.464^**^	.389^*^	.611^*^

All values are variance explained (pseudo-R^2^); Health care utilization (dependent) variables refer to utilization during the follow-up period, whereas independent variables refer to measurements taken before the follow-up period

^*^p < 0.05, ^**^p < 0.01

### Individual predictors of healthcare utilization

Parsimonious model results were used to identify significant individual predictors of health care utilization. Results of the parsimonious models are listed in Table 6 and summarized below. Variance explained (pseudo-R^2^) values reported in the summaries below are specific to performance of individual parsimonious models, and are different than pseudo-R^2^ values of the full multivariate models listed in Table 5. Comparison of adjusted odds ratios (OR) and 95% confidence interval (CI) were used to determine the relative strength of each predictor in the parsimonious models. Odds ratios are reported from the parsimonious models as these are intuitive, clinically interpretable predictive indices. Summary findings for all utilization outcomes are presented in Table 7. In summarizing results, we focus on predictors that were identified consistently across complete case and weighted analytic models for each type of healthcare service.

**Table 6:** Parsimonious multivariate logistic regression model results for prediction of any care and individual service utilization (*n* = 246) ^a^

Dependent variable	Independent variable	*Complete case*					*Inverse probability of attrition weighted*				
		B	S.E.	Sig.	Odds ratio	Pseudo R^2^	B	S.E.	Sig.	Odds ratio	Pseudo R^2^
Any care	Chronicity	0.79	0.38	0.04	2.19	0.30	0.73	0.41	0.07	2.08	0.31
	Baseline pain	0.37	0.12	0.002	1.45						
	CCI	0.21	0.12	0.07	1.23		0.31	0.14	0.03	1.36	
	Baseline disability	0.39	0.20	0.05	1.48		0.90	0.19	< 0.001	2.47	
	Change in pain	0.37	0.11	< 0.001	1.45		0.25	0.10	0.02	1.28	
Opioids	Surgery for condition					0.33	0.95	0.49	0.51	2.58	0.36
	Chronicity	1.03	0.59	0.08	2.80						
	CCI	0.43	0.13	< 0.001	1.54		0.47	0.14	< 0.001	1.60	
	Baseline pain	0.53	0.13	< 0.001	1.70		0.57	0.15	< 0.001	1.76	
	Baseline 10-item OSPRO-YF						0.01	0.03	0.96	1.00	
	Change in pain	0.54	0.14	< 0.001	1.71		0.53	0.15	< 0.001	1.70	
	Additional OSPRO-YF 7-items (baseline)	−0.09	0.05	0.08	0.92		−0.01	0.05	0.07	0.91	
Injection	Race (non-white) ^b^	−1.79	1.05	0.09	0.17	0.22	−2.15	1.19	0.07	0.12	0.24
	Chronicity	1.27	0.65	0.05	3.57		1.31	0.73	0.07	3.71	
	Baseline disability	0.76	0.20	< 0.01	2.14		0.76	0.21	< 0.01	2.14	
Surgery	CCI					0.30	0.15	0.10	0.15	1.16	0.34
	Baseline disability	1.14	0.28	< 0.001	3.13		1.18	0.31	< 0.001	3.25	
	Change in 10-item OSPRO-YF score	0.12	0.05	0.03	1.12		0.13	0.06	0.02	1.14	
	Change in disability	1.11	0.42	0.01	3.04		1.12	0.44	0.01	3.05	
Diagnostic tests or imaging	Chronicity	0.60	0.43	0.16	1.82	0.29					0.33
	CCI	0.30	0.12	0.02	1.35		0.37	0.13	< 0.001	1.45	
	Baseline disability	0.81	0.18	< 0.001	2.25		0.98	0.19	< 0.001	2.66	
	Change in pain	0.34	0.11	< 0.001	1.41		0.37	0.11	< 0.001	1.45	
Emergency room	Age	−0.09	0.03	< 0.001	0.91	0.48	−0.06	0.03	0.03	0.94	0.50
	Anatomical location (neck) ^c^	0.54	0.97	0.58	1.71		−0.37	0.92	0.69	0.69	
	Anatomical location (low back) ^c^	0.72	0.95	0.45	2.05		−0.04	0.89	0.97	0.96	
	Anatomical location (shoulder) ^c^	−2.86	1.49	0.06	0.06		−3.52	1.53	0.02	0.03	
	Insurance (public) ^d^	1.89	1.06	0.08	6.65		1.81	1.11	0.10	6.11	
	Insurance (other) ^d^	2.20	0.88	0.01	8.99		2.58	0.86	< 0.001	13.15	
	Chronicity	1.76	1.26	0.16	5.81						
	Surgery for condition	1.69	0.94	0.07	5.43		1.77	0.93	0.06	5.86	
	Baseline disability	1.59	0.47	< 0.001	4.88		1.20	0.34	< 0.001	3.33	
	Additional OSPRO-YF 7-items (baseline)	−0.20	0.09	0.03	0.82						
	Change in 10-item OSPRO-YF score						0.14	0.07	0.05	1.15	
	Change in pain	0.57	0.22	0.01	1.77		0.46	0.21	0.03	1.59	

*CCI* Charlson comorbidity index, *OSPRO-YF* Pain-related psychological distress screening tool

^a^ Health care utilization (dependent) variables refer to utilization during the follow-up period, whereas independent variables refer to measurements taken before the follow-up period

^b^ Reference value is White

^c^ Reference value is knee

^d^ Reference value is private insurance

**Table 7:** Summary of consistent individual predictors for each utilization outcome ^*^

Dependent variable	Utilization outcome					
	Any care	Opioids	Injection	Surgery	Diagnostic tests or imaging	Emergency room
Age						X
Insurance						X
Comorbidities (CCI)		X			X	
Baseline disability	X		X	X	X	X
Baseline pain		X				
Change in pain	X	X			X	X
Change in disability				X		
Change in 10-item OSPRO-YF				X		

*CCI* Charlson comorbidity index, *OSPRO-YF* Pain-related psychological distress screening tool

^*^Significant predictors (*p* < .05) for each dependent variable denoted with “X”

### Any healthcare

The final parsimonious models for any healthcare utilization differed slightly between complete case and weighted analyses (Table 6). Included in the models were chronicity of symptoms, CCI, baseline pain, baseline disability, and change in pain from baseline to 4-week follow-up. However, only baseline disability (OR = 1.48–2.47, *p* < 0.05) and change in pain (OR = 1.28–1.45, *p* < 0.05) were significant predictors in both models, with greater baseline disability and worsening pain associated with higher odds of any utilization.

### Utilization of individual services

#### Opioids

Comorbidity index score (i.e. CCI), baseline pain and change in pain were consistent predictors between the models of opioid utilization. In these models, higher pain (OR = 1.70–1.76, *p* < 0.001), CCI (OR = 1.54–1.60, *p* < 0.001) and increase in pain (OR = 1.70–1.71, *p* < 0.001) were associated with higher odds of opioid utilization. These models explained approximately 30% of the variation in opioid use.

#### Injection

A combination of race, chronicity and baseline disability explained slightly more than 20% of the variance in injection utilization, however the only significant individual predictor was baseline disability. In both models, each 1-standard deviation increase in disability was associated with approximately twice the odds of receiving injection (OR = 2.14, *p* < .01). The pseudo-R^2^ value for these models was lowest of all specific healthcare services, suggesting injection may be the most difficult service to predict with the included variable set.

#### Surgery

Baseline disability (OR = 3.13–3.25, *p* < 0.001), change in disability (OR = 3.04–3.05, *p* = 0.01) and change in 10-item OSPRO-YF score (OR = 1.12–1.14, *p* < 0.05) where consistent predictors of subsequent surgery. Notably, magnitude of prediction was comparable between change in disability and baseline disability. This was the only parsimonious model to include an OSPRO tool. In this case, an increase in pain-related psychological distress measured by the OSPRO-YF 10-item questionnaire over the first 4 weeks was associated with higher odds of surgery. The 3 predictors in this model explained just over 30% of the variance in surgery utilization.

#### Diagnostic tests or imaging

Comorbidity index score (OR = 1.35–1.45, *p* < 0.05), baseline disability (OR = 2.25–2.66, *p* < 0.001), and baseline to 4-week change in pain intensity (OR = 3.04–3.05, *p* = 0.01) were significant predictors of diagnostic test or imaging utilization. Among these, baseline disability was the strongest predictor. In these models, higher comorbidity index, higher baseline disability and worsening pain were associated with higher odds of utilization. Together, these variables explained approximately 30% of the variance in utilization.

#### Emergency room

Models for emergency room use had the highest pseudo-R^2^ values of any individual service (0.48–0.50), but also had the largest number of predictors (8–9). Agreement between complete case and weighted models was moderate. The models converged on the following predictors: age (OR = 0.91–0.94, *p* < 0.05), insurance (OR = 8.99–13.15, *p* < 0.05), baseline disability (OR = 3.33–4.88, *p* < 0.001), and change in pain (OR = 1.59–1.77, *p* < 0.05). Higher utilization was associated with younger age, other insurance (e.g., self-pay, Worker’s Compensation, or other commercial insurance) compared to private insurance, higher baseline disability and worsening of pain. In the weighted analysis, subjects with knee pain were less likely to utilize the emergency room than those with low back pain. However, this relationship was not significant (*p* = .06) in the complete case analysis. Of the significant predictors in both models, insurance status was the strongest individual predictor of subsequent emergency room use.

## Discussion

This study identified novel predictors for pain-related utilization outcomes following an episode of physical therapy for a primary complaint of musculoskeletal pain. The most robust finding from these analyses was that baseline disability and change in pain intensity over the first 4 weeks following physical therapy evaluation were consistent predictors of subsequent pain-related healthcare utilization in those participants that completed all follow up. Aside from these robust predictors, other individual predictors of utilization were highly outcome-specific. High model specificity for utilization outcomes observed in this study is consistent with a recent systematic review that found similar levels of model specificity for more traditional outcomes like pain intensity, disability and work absenteeism [14]. Across models, health-related variables were generally stronger predictors than sociodemographic factors, which is also supported by prior research [15, 16]. Additionally, there were cases when prediction models were improved for specific services (e.g. surgery, use of opioids) when considering change in pain, disability or pain-related psychological distress. A notable finding is that the OSPRO-YF had the greatest utility when used to measure change in pain-related psychological distress. Current risk prediction paradigms in musculoskeletal pain consider only baseline pain-related psychological distress. However, these results underscore the importance of routine pain-related psychological distress monitoring throughout the early phases of rehabilitation especially if the goal is to identify risk for subsequent pain-related healthcare utilization. The implications of these collective findings are that treatment pathways may provide greater value by 1) addressing modifiable health-related variables like pain, disability and pain-related psychological distress, 2) routine monitoring of these health-related variables and 3) offering treatment alternatives that safely escalate care if needed while minimizing risk of harm and unhelpful utilization.

Opioids and diagnostic tests and imaging were the two most common subsequent healthcare services utilized following physical therapy. Of the individuals that completed follow up and had any subsequent healthcare utilization, approximately 42% reported opioid use and 70% reported use of diagnostic tests and imaging. An important health-related predictor of these services was level of comorbidity burden. For those with high comorbidity burden and inadequate treatment response to physical therapy, use of additional diagnostic tests and imaging or low-dose opioids may be appropriate in some cases. But given the growing public health concern over opioid use and the desire to avoid unnecessary treatment driven by imaging, our results suggest the importance of considering disease burden when developing treatment pathways and healthcare policy to mitigate risk for avoidable use of these services. Interestingly, neither versions of the OSPRO-ROS predicted utilization outcomes even though it has been linked to mental health, comorbidity, and persistent pain state in other analyses [20, 21]. Systemic symptom burden is a measure of patient complexity that is related to but distinct from comorbidity burden [36, 47]. In these analyses, the chronic condition measure (i.e. the CCI) was a better predictor of utilization than symptom burden (i.e. OSPRO-ROS). The reasons for this finding are unclear but may be related to providers and patients being more likely to pursue follow-up medical care for musculoskeletal pain when known co-existing conditions are present as opposed to reporting of symptoms alone. The distinction between symptom and disease burden in defining musculoskeletal patient complexity, and its influence on clinical decision-making and outcomes, should be the subject of future research particularly related to aging populations [48].

Utilization outcomes benchmarks have not been established to determine how the percentage of subsequent healthcare use in this study compares to outcomes using other health services. Prior studies suggest physical therapy is associated with reduced incidence of additional healthcare use compared to not using physical therapy in patients with acute low back pain [10, 49]. Some additional healthcare use is expected following physical therapy, especially among individuals that are on long-term pain management pathways due to chronic or persistent symptoms. Yet with over 40% reporting subsequent pain-related healthcare among those completing follow-up, it is apparent that opportunities exist to improve pathway selection and/or the effectiveness of physical therapy for individuals with musculoskeletal pain. This finding is particularly notable given recent efforts to define physical therapy as an effective first line, non-pharmacological treatment option against more invasive or higher risk services, such as surgery or opioid use, respectively. Predictive variables identified in this analysis can be used to develop risk models that better inform pathway selection for those seeking physical therapy for musculoskeletal pain. The precise application of these risk models, and how they inform policy and practice should be the target of future study. However, physical therapy re-design might incorporate enhanced treatment monitoring to assess ongoing risk for downstream utilization, as well as physical therapist-led interventions to more thoroughly address important modifiable factors such as pain intensity, disability and pain-related psychological distress [38]. Improved pathway selection might entail the consideration of referral to or co-treatment with other providers to more adequately address non-modifiable characteristics. Collectively, these approaches could improve the value of physical therapy by minimizing risk for high downstream healthcare utilization and potentially unwarranted escalation of care.

The primary strength of the study is longitudinal follow-up at multiple time points following an episode of physical therapy for a variety of musculoskeletal pain conditions. Anatomical location of pain was not a significant predictor of healthcare use in all but one model, suggesting results are widely applicable across a spectrum of musculoskeletal pain conditions. Another strength of this cohort study is the assessment of various healthcare utilization outcomes of interest for establishing health policy. When considered alongside more traditional pain- or disability-related outcomes prediction models, these findings will improve the ability of healthcare systems and providers to make decisions in value-based purchasing environments. The consideration of multiple screening tools (i.e. yellow flags and review of systems) and treatment monitoring variables is also a strength of this study as screening and systematic treatment monitoring are not routine in clinical practice. A final strength is inclusion of multiple sociodemographic, health-related and psychosocial factors as potential predictors. Healthcare outcomes and utilization exhibit emergent properties that require the consideration of multiple, competing factors to fully explain [50]. However, explained variance estimates in our models ranged from 34 to 61%, suggesting further research is necessary to identify additional factors contributing to healthcare utilization following physical therapy.

The primary limitation of the study is the high number of subjects lost to follow-up. We attempted to account for the bias introduced by loss to follow-up in our models with IPAW, which is a robust strategy for conducting analyses with missing data [41, 51]. We observed good concordance between results of complete case and weighted analyses, giving us confidence in our findings. However, important differences in age, race, education, symptom onset, baseline pain intensity, and baseline pain-related psychological distress were noted between those who did and did not complete follow-up. These differences suggest that the group lost to follow-up may represent a unique population to whom these results may not apply. Different factors may predict utilization outcomes for this unique population. As a result, readers should exercise caution when extending these findings to individuals and populations that substantially differ from the analytic sample in this study. Specifically, these predictive models may need to be adjusted for younger individuals of non-white race, with lower education levels, sudden onset of symptoms, and those with higher pain intensity and pain-associated distress.

A second limitation is that we did not know about the subjects’ prior experiences with physical therapy, or whether they arrived at physical therapy through direct access or referral from another provider. These factors could be associated with treatment expectations, which have known effects on treatment outcomes [52, 53]. We also did not collect specific information on treatment. But by including changes in pain, disability, and pain-related psychological distress in the models, we were able to account for treatment response. The benefit of this approach is that models are generalizable for predicting utilization outcomes across “real-world” pragmatic physical therapy settings where treatment variation is expected. The drawback is that we are prohibited from making conclusions regarding which characteristics of the clinical encounter might influence subsequent pain-related healthcare utilization. Important characteristics to consider would include number of visits, type of interventions or whether patients completed their course of physical therapy. These have been proposed or identified as important contributors to downstream costs following physical therapy [54, 55] and may be a source of unexplained variance in our models. Characteristics of the clinical encounter should be considered in future studies to refine the prediction models developed in our analyses.

Third, we were unable to adequately model the specific effects of worker’s compensation, self-pay and some commercial insurance coverage on utilization due to the low incidence of these forms of payment in our study sample. Modeling these separately would have created the potential for unreliable and imprecise effect estimates. Readers should consider the within-group heterogeneity caused by this approach and exercise caution when applying these results to individuals who do not have traditional public or private insurance coverage. Future studies should investigate the performance of the OSPRO tools in predicting outcomes for patients with Worker’s Compensation.

A final limitation is the use of patient recall to measure utilization. To mitigate recall bias, we used two follow-up points, at 6 and 12 months. However, under- or over-reporting of utilization is often a concern with studies requiring subject recall [56–58]. Medical record and claims data were not available for these subjects. Readers should consider our inability to independently confirm utilization when interpreting results.

In future studies, we will embed the OSPRO tools into electronic medical record (EMR) databases to refine and test outcomes prediction models at the health care systems level. Importantly, we will collect clinical encounter data through the EMR and combine it with administrative or billing data to confirm the results of this study with more objective measures of health care use. These studies will also allow us to provide better guidance on how to use the OSPRO tools to identify serious psychiatric involvement or systemic sources of pain that require medical referral. Finally, we will explore alternative scoring strategies for the tools, such as weighted scoring for the OSPRO-ROS and use of predicted full-length psychological questionnaire scores for the OSPRO-YF. Healthcare providers could then use the collective information from these studies to build learning health systems that facilitate effective, real-time clinical decision-making support to improve value of care for patients with musculoskeletal pain.

## Conclusion

Baseline disability and change in pain intensity were important predictors of any subsequent pain-related healthcare utilization, while predictors of individual service utilization were outcome-specific. Identification of risk is improved through treatment monitoring for pain and, in some cases, disability and pain-related psychological distress. Comorbidity burden was an important predictor of subsequent utilization of opioids and diagnostic tests and imaging, both of which have been recent targets of healthcare policy to constrain their unnecessary use. Future research is needed to refine these predictor variables and incorporate them into risk models that support clinical decision-making so that treatment effectiveness and efficiency are optimized in value-based systems.

## Abbreviations

CCI: Charlson comorbidity index
OSPRO: Optimal Screening for Prediction of Referral and Outcome
OSPRO-ROS: Review of systems screening tool from OSPRO cohort study
OSPRO-YF: Pain-related psychological distress screening tool from OSPRO cohort study

## References

[1] Von Korff M, Scher AI, Helmick C, Carter-Pokras O, Dodick DW, Goulet J (2016). United states national pain strategy for population research: concepts, definitions, and pilot data. J Pain Off J Am Pain Soc.

[2] Clarke JL, Skoufalos A, Scranton R (2016). The American opioid epidemic: population health implications and potential solutions. Report from the national stakeholder panel. Popul Health Manag.

[3] Dowell D, Haegerich TM, Chou R (2016). CDC guideline for prescribing opioids for chronic pain--United States, 2016. JAMA.

[4] Boyles R, Toy P, Mellon J, Hayes M, Hammer B (2011). Effectiveness of manual physical therapy in the treatment of cervical radiculopathy: a systematic review. J Man Manip Ther.

[5] Bürge E, Monnin D, Berchtold A, Allet L (2016). Cost-effectiveness of physical therapy only and of usual care for various health conditions: systematic review. Phys Ther.

[6] Deyle GD, Allison SC, Matekel RL, Ryder MG, Stang JM, Gohdes DD (2005). Physical therapy treatment effectiveness for osteoarthritis of the knee: a randomized comparison of supervised clinical exercise and manual therapy procedures versus a home exercise program. Phys Ther.

[7] Deyle GD, Henderson NE, Matekel RL, Ryder MG, Garber MB, Allison SC (2000). Effectiveness of manual physical therapy and exercise in osteoarthritis of the knee. A randomized, controlled trial. Ann Intern Med.

[8] Freburger JK, Carey TS, Holmes GM (2006). Effectiveness of physical therapy for the management of chronic spine disorders: a propensity score approach. Phys Ther.

[9] Kuhn JE, Dunn WR, Sanders R, An Q, Baumgarten KM, Bishop JY (2013). Effectiveness of physical therapy in treating atraumatic full-thickness rotator cuff tears: a multicenter prospective cohort study. J Shoulder Elb Surg.

[10] Fritz JM, Childs JD, Wainner RS, Flynn TW (2012). Primary care referral of patients with low back pain to physical therapy: impact on future health care utilization and costs. Spine.

[11] Fritz JM, Brennan GP, Hunter SJ, Magel JS (2013). Initial management decisions after a new consultation for low back pain: implications of the usage of physical therapy for subsequent health care costs and utilization. Arch Phys Med Rehabil.

[12] Hill JC, Dunn KM, Lewis M, Mullis R, Main CJ, Foster NE (2008). A primary care back pain screening tool: identifying patient subgroups for initial treatment. Arthritis Rheum.

[13] Traeger AC, Henschke N, Hübscher M, Williams CM, Kamper SJ, Maher CG (2016). Estimating the risk of chronic pain: development and validation of a prognostic model (PICKUP) for patients with acute low back pain. PLoS Med.

[14] Karran EL, McAuley JH, Traeger AC, Hillier SL, Grabherr L, Russek LN (2017). Can screening instruments accurately determine poor outcome risk in adults with recent onset low back pain? A systematic review and meta-analysis. BMC Med.

[15] Azevedo LF, Costa-Pereira A, Mendonça L, Dias CC, Castro-Lopes JM (2013). Chronic pain and health services utilization: is there overuse of diagnostic tests and inequalities in nonpharmacologic treatment methods utilization?. Med Care.

[16] Langley P, Müller-Schwefe G, Nicolaou A, Liedgens H, Pergolizzi J, Varrassi G (2010). The societal impact of pain in the European Union: health-related quality of life and healthcare resource utilization. J Med Econ.

[17] Pérez C, Navarro A, Saldaña MT, Wilson K, Rejas J (2015). Modeling the predictive value of pain intensity on costs and resources utilization in patients with peripheral neuropathic pain. Clin J Pain.

[18] Hill JC, Fritz JM (2011). Psychosocial influences on low back pain, disability, and response to treatment. Phys Ther.

[19] George SZ, Beneciuk JM, Lentz TA, Wu SS (2017). The Optimal Screening for Prediction of Referral and Outcome (OSPRO) in patients with musculoskeletal pain conditions: a longitudinal validation cohort from the USA. BMJ Open.

[20] George SZ, Beneciuk JM, Lentz TA, Wu SS, Dai Y, Bialosky JE, Zeppieri G Jr. Optimal Screening for Prediction of Referral and Outcome (OSPRO) for Musculoskeletal Pain Conditions: Results From the Validation Cohort. J Orthop Sports Phys Ther. 2018;48(6):460–75. 10.2519/jospt.2018.7811 PMC6053060 29629615

[21] Beneciuk JM, Lentz TA, He Y, Wu SS, George SZ (2018). Prediction of persistent musculoskeletal pain at 12 months: a secondary analysis of the Optimal Screening for Prediction of Referral and Outcome (OSPRO) validation cohort study. Phys Ther.

[22] Freburger JK, Holmes GM, Agans RP, Jackman AM, Darter JD, Wallace AS (2009). The rising prevalence of chronic low back pain. Arch Intern Med.

[23] Carey TS, Freburger JK, Holmes GM, Jackman A, Knauer S, Wallace A (2010). Race, care seeking, and utilization for chronic back and neck pain: population perspectives. J Pain Off J Am Pain Soc.

[24] Jensen MP, Turner JA, Romano JM, Fisher LD (1999). Comparative reliability and validity of chronic pain intensity measures. Pain.

[25] Bolton JE (1999). Accuracy of recall of usual pain intensity in back pain patients. Pain.

[26] Childs JD, Piva SR, Fritz JM (2005). Responsiveness of the numeric pain rating scale in patients with low back pain. Spine.

[27] Vernon H (2008). The neck disability index: state-of-the-art, 1991-2008. J Manip Physiol Ther.

[28] Vernon H, Mior S (1991). The neck disability index: a study of reliability and validity. J Manip Physiol Ther.

[29] Hudson-Cook N, Tomes-Nicholson K, Breen A, Roland M, Jenner J (1989). A revised Oswestry disability questionnaire. Back pain: new approaches to rehabilitation and education.

[30] Fritz JM, Irrgang JJ (2001). A comparison of a modified Oswestry low back pain disability questionnaire and the Quebec back pain disability scale. Phys Ther.

[31] Beaton DE, Wright JG, Katz JN, Upper Extremity Collaborative Group (2005). Development of the QuickDASH: comparison of three item-reduction approaches. J Bone Joint Surg Am.

[32] Irrgang JJ, Anderson AF, Boland AL, Harner CD, Kurosaka M, Neyret P (2001). Development and validation of the international knee documentation committee subjective knee form. Am J Sports Med.

[33] Butera KA, Lentz TA, Beneciuk JM, George SZ (2016). Preliminary evaluation of a modified STarT back screening tool across different musculoskeletal pain conditions. Phys Ther.

[34] Charlson ME, Pompei P, Ales KL, MacKenzie CR (1987). A new method of classifying prognostic comorbidity in longitudinal studies: development and validation. J Chronic Dis.

[35] Katz JN, Chang LC, Sangha O, Fossel AH, Bates DW (1996). Can comorbidity be measured by questionnaire rather than medical record review?. Med Care.

[36] George SZ, Beneciuk JM, Bialosky JE, Lentz TA, Zeppieri G, Pei Q (2015). Development of a review-of-systems screening tool for orthopaedic physical therapists: results from the Optimal Screening for Prediction of Referral and Outcome (OSPRO) cohort. J Orthop Sports Phys Ther.

[37] Lentz TA, Beneciuk JM, Bialosky JE, Zeppieri G, Dai Y, Wu SS (2016). Development of a yellow flag assessment tool for orthopaedic physical therapists: results from the Optimal Screening for Prediction of Referral and Outcome (OSPRO) cohort. J Orthop Sports Phys Ther.

[38] Beneciuk JM, Fritz JM, George SZ (2014). The STarT back screening tool for prediction of 6-month clinical outcomes: relevance of change patterns in outpatient physical therapy settings. J Orthop Sports Phys Ther.

[39] Myers RH (2000). Classical and modern regression with applications.

[40] Weuve J, Tchetgen Tchetgen EJ, Glymour MM, Beck TL, Aggarwal NT, Wilson RS (2012). Accounting for bias due to selective attrition: the example of smoking and cognitive decline. Epidemiol Camb Mass.

[41] Hernán MA, Hernández-Díaz S, Robins JM (2004). A structural approach to selection bias. Epidemiol Camb Mass.

[42] Kent P, Keating JL, Leboeuf-Yde C (2010). Research methods for subgrouping low back pain. BMC Med Res Methodol.

[43] Peduzzi P, Concato J, Kemper E, Holford TR, Feinstein AR (1996). A simulation study of the number of events per variable in logistic regression analysis. J Clin Epidemiol.

[44] Tabachnick BG, Fidell LS (2006). Using multivariate statistics.

[45] Green SB (1991). How many subjects does it take to do a regression analysis. Multivar Behav Res.

[46] Harris RJ (2001). A primer of multivariate statistics.

[47] Piette JD, Kerr EA (2006). The impact of comorbid chronic conditions on diabetes care. Diabetes Care.

[48] Rice ASC, Smith BH, Blyth FM (2016). Pain and the global burden of disease. Pain.

[49] Fritz JM, Cleland JA, Speckman M, Brennan GP, Hunter SJ (2008). Physical therapy for acute low back pain: associations with subsequent healthcare costs. Spine.

[50] Lentz TA, Harman JS, Marlow NM, George SZ (2017). Application of a value model for the prevention and management of chronic musculoskeletal pain by physical therapists. Phys Ther.

[51] Sterne JAC, White IR, Carlin JB, Spratt M, Royston P, Kenward MG (2009). Multiple imputation for missing data in epidemiological and clinical research: potential and pitfalls. BMJ.

[52] Bishop MD, Mintken PE, Bialosky JE, Cleland JA (2013). Patient expectations of benefit from interventions for neck pain and resulting influence on outcomes. J Orthop Sports Phys Ther.

[53] Bialosky JE, Bishop MD, Cleland JA (2010). Individual expectation: an overlooked, but pertinent, factor in the treatment of individuals experiencing musculoskeletal pain. Phys Ther.

[54] Hanney WJ, Masaracchio M, Liu X, Kolber MJ (2016). The influence of physical therapy guideline adherence on healthcare utilization and costs among patients with low back pain: a systematic review of the literature. PLoS One.

[55] Childs JD, Fritz JM, Wu SS, Flynn TW, Wainner RS, Robertson EK, et al. Implications of early and guideline adherent physical therapy for low back pain on utilization and costs. BMC Health Serv Res. 2015;15 10.1186/s12913-015-0830-3. 10.1186/s12913-015-0830-3 PMC4393575 25880898

[56] Yu S-T, Chang H-Y, Lin M-C, Lin Y-H (2009). Agreement between self-reported and health insurance claims on utilization of health care: a population study. J Clin Epidemiol.

[57] Petrou S, Murray L, Cooper P, Davidson LL (2002). The accuracy of self-reported healthcare resource utilization in health economic studies. Int J Technol Assess Health Care.

[58] Short ME, Goetzel RZ, Pei X, Tabrizi MJ, Ozminkowski RJ, Gibson TB (2009). How accurate are self-reports? Analysis of self-reported health care utilization and absence when compared with administrative data. J Occup Environ Med.

